# Neuroprotective and anti-epileptic potentials of genus Artemisia L.

**DOI:** 10.3389/fphar.2022.1021501

**Published:** 2022-10-19

**Authors:** Bayan Sailike, Zhannur Omarova, Janar Jenis, Altynay Adilbayev, Burkitkan Akbay, Sholpan Askarova, Wei-Lin Jin, Tursonjan Tokay

**Affiliations:** ^1^ Biology Department, School of Sciences and Humanities, Nazarbayev University, Nur-Sultan, Kazakhstan; ^2^ Research Center for Medicinal Plants of Al-Farabi Kazakh National University, Almaty, Kazakhstan; ^3^ Department of Biomedical Sciences, Nazarbayev University School of Medicine, Nur-Sultan, Kazakhstan; ^4^ Laboratory of Bioengineering and Regenerative Medicine, National Laboratory of Astana, Nazarbayev University, Nur-Sultan, Kazakhstan; ^5^ Institute of Cancer Neuroscience, Medical Frontier Innovation Research Center, The First Hospital of Lanzhou University, The First Clinical Medical College of Lanzhou University, Lanzhou, China

**Keywords:** epilepsy, excitotoxicity, oxidative stress, neuroinflammation, genus artemisia, neuroprotection, cognitive impairment

## Abstract

The Genus Artemisia L. is one of the largest genera in the Asteraceae family growing wild over in Europe, North America, and Central Asia and has been widely used in folk medicine for the treatment of various ailments. Phytochemical and psychopharmacological studies indicated that the genus Artemisia extracts contain various antioxidant and anti-inflammatory compounds and possess antioxidant, anti-inflammatory, antimicrobial, antimalarial, and antitumor activity. Recently, increasing experimental studies demonstrated that many Artemisia extracts offer a great antiepileptic potential, which was attributed to their bioactive components *via* various mechanisms of action. However, detailed literature on the antiepileptic properties of the genus Artemisia and its mechanism of action is segregated. In this review, we tried to gather the detailed neuroprotective and antiepileptic properties of the genus Artemisia and its possible underlying mechanisms. In this respect, 63 articles were identified in the PubMed and Google scholars databases, from which 18 studies were examined based on the pharmacological use of the genus Artemisia species in epilepsy. The genus Artemisia extracts have been reported to possess antioxidant, anti-inflammatory, neurotransmitter-modulating, anti-apoptotic, anticonvulsant, and pro-cognitive properties by modulating oxidative stress caused by mitochondrial ROS production and an imbalance of antioxidant enzymes, by protecting mitochondrial membrane potential required for ATP production, by upregulating GABA-A receptor and nACh receptor activities, and by interfering with various anti-inflammatory and anti-apoptotic signaling pathways, such as mitochondrial apoptosis pathway, ERK/CREB/Bcl-2 pathway and Nrf2 pathway. This review provides detailed information about some species of the genus Artemisia as potential antiepileptic agents. Hence, we recommend further investigations on the purification and identification of the most biological effective compounds of Artemisia and the mechanisms of their action to cure epilepsy and other neurological diseases.

## 1 Introduction

Epilepsy is a type of neurological disorder characterized by symptoms of periodic, unpredictable, and recurrent seizures (Beghi et al., 2019). According to the International League Against Epilepsy (ILAE) classification in 2017, epilepsies are classified as focal, generalized, combined generalized and focal epilepsy or unknown types (Scheffer et al., 2017). Although the cause of epilepsy is still unknown in most cases, seizures can be caused by any damage associated with metabolic disorders, infectious diseases, genetic mutation, or immune disorders that disrupt brain function (Devinsky et al., 2018).

Currently available antiepileptic drugs (AEDs) are only partially efficient for epilepsy. Although AEDs control nearly 70% of patients with epilepsy, 5%–10% of patients still require additional medications and more than 20% of patients continue to have seizures after treatment. They can only treat symptoms of epilepsy without making any significant progress in neither reversing its underlying mechanisms nor offering neuroprotection caused by the pathogenesis of epilepsy, resulting in about 15%–40% of all patients with epilepsy, unfortunately, continuing to have seizures that impair their daily living (Sankaraneni and Lachhwani, 2015). Moreover, most AEDs have shown cognitive, psychiatric, and behavioral side effects on patients (Chen et al., 2017). For example, cognitive abnormality, especially impairment in learning and memory, is one of the serious comorbidities caused by epileptic seizures and/or AEDs that has a significant negative impact on patients’ quality of life (Holmes, 2015). The neuronal damage and death caused by seizure-associated excitotoxicity are most likely responsible for both recurrent epilepsy itself and epilepsy-induced cognitive impairment. Therefore, dampening excitotoxicity, which is resulted from an imbalance between the excitatory and inhibitory neurotransmitters, was the main target of currently available AEDs (Sankaraneni and Lachhwani, 2015).

Since the current anti-epileptic treatment is not ideal, the development of more effective and clinically relevant novel therapeutic approaches for epilepsy is an urgent need and hope. In recent years, more and more evidence has shown that oxidative stress and neuroinflammation play important roles in the pathophysiology of acquired epilepsy (Vezzani, Balosso, and Ravizza, 2019). In this respect, oxidative stress and neuroinflammation pathways could be desirable therapeutic targets in epilepsy. Previous studies have demonstrated that the use of plant extracts and plant-based antioxidant and anti-inflammatory compounds such as flavonoids could reduce or eliminate neuroinflammation and oxidative damage (Kwon et al., 2019). One such well-studied herbal plant family is the genus Artemisia. Many phytochemical and pharmacological studies have reported that the genus Artemisia contains a huge amount of interesting polyphenolic constituents and showed promising anticonvulsant properties in experimental studies using various animal models of epilepsy through various mechanistic pathways. However, the detailed literature on the antiepileptic properties of the genus Artemisia and its mechanism of action is segregated. In this review, we tried to gather the detailed neuroprotective and antiepileptic properties of the genus Artemisia and its possible underlying mechanisms.

In this mini-review, we have explored the therapeutic potential of Artemisia for the treatment of epilepsy. Data were collected by searching relevant original articles using the scientific databases Pubmed and Google scholars with the keywords “artemisia,” “neuroprotection,” “anticonvulsants” or “anti-epileptic drugs.” As a result, 63 articles were retrieved, of which 18 original research articles were reviewed based on the pharmacological use of Artemisia in epilepsy. This review discusses current research on certain species of the genus Artemisia with anti-epileptic and neuroprotective activities. In addition, the anti-epileptic mechanisms of their bioactive components and the experimental models used in studies are also introduced and discussed.

## 2 The redox-associated neural apoptosis and its role in epilepsy

Oxidative Stress (OS) might play a role in epilepsy and associated cognitive disorders (Geronzi, Lotti and Grosso, 2018). A growing number of studies in cellular and animal models have shown that OS is involved in the development of recurrent seizures, status epilepticus (SE), and learning abnormalities by causing neural damage and death (Vilar Da Fonsêca et al., 2019; Eastman, D’Ambrosio and Ganesh, 2020; Lin et al., 2020; Golub and Reddy, 2022). However, the exact mechanisms by which how OS can provoke a normal brain to develop spontaneous seizures and cognitive comorbidities have not been completely elucidated. Some experimental evidence revealed an association between neuronal apoptosis and epileptogenesis whilst it is unclear whether apoptosis is a cause or a consequence of epilepsy.

Apoptosis is primarily induced by two main pathways—the extrinsic and intrinsic pathways. In the extrinsic pathway, apoptosis is initiated by the activation of death receptors, whereas, in the intrinsic pathway, it is induced by the activation of a series of apoptotic protein that changes mitochondrial permeability, leading to the release of cytochrome c into the cytosol (Bedoui, Herold and Strasser, 2020). The Bcl-2 family is a well-characterized protein family involved in the modulation of apoptotic cell death, consisting of anti-apoptotic Bcl-2 protein and pro-apoptotic Bax and Bak, which are key modulators of the intrinsic (mitochondrial) pathway of apoptosis. Under normal conditions, Bcl-2, which localizes to the outer mitochondrial membrane, promotes cell survival by inhibiting Bax/Bak oligomerization, which would otherwise facilitate the release of cytochrome c from mitochondria (Ge and Wang, 2020). In response to cellular stress, Bax/Bak oligomers create pores in the mitochondrial outer membrane, leading to depolarization of the mitochondrial membrane potential (ΔΨm) and release of cytochrome c into the cytoplasm (Ly, Grubb and Lawen, 2003; Westphal et al., 2011). Cytosolic cytochrome c then triggers apoptosis by binding to Apaf-1 (apoptotic protease activating factor 1) and procaspase-9 that further activates downstream caspases (Westphal et al., 2011).

**FIGURE 1:**
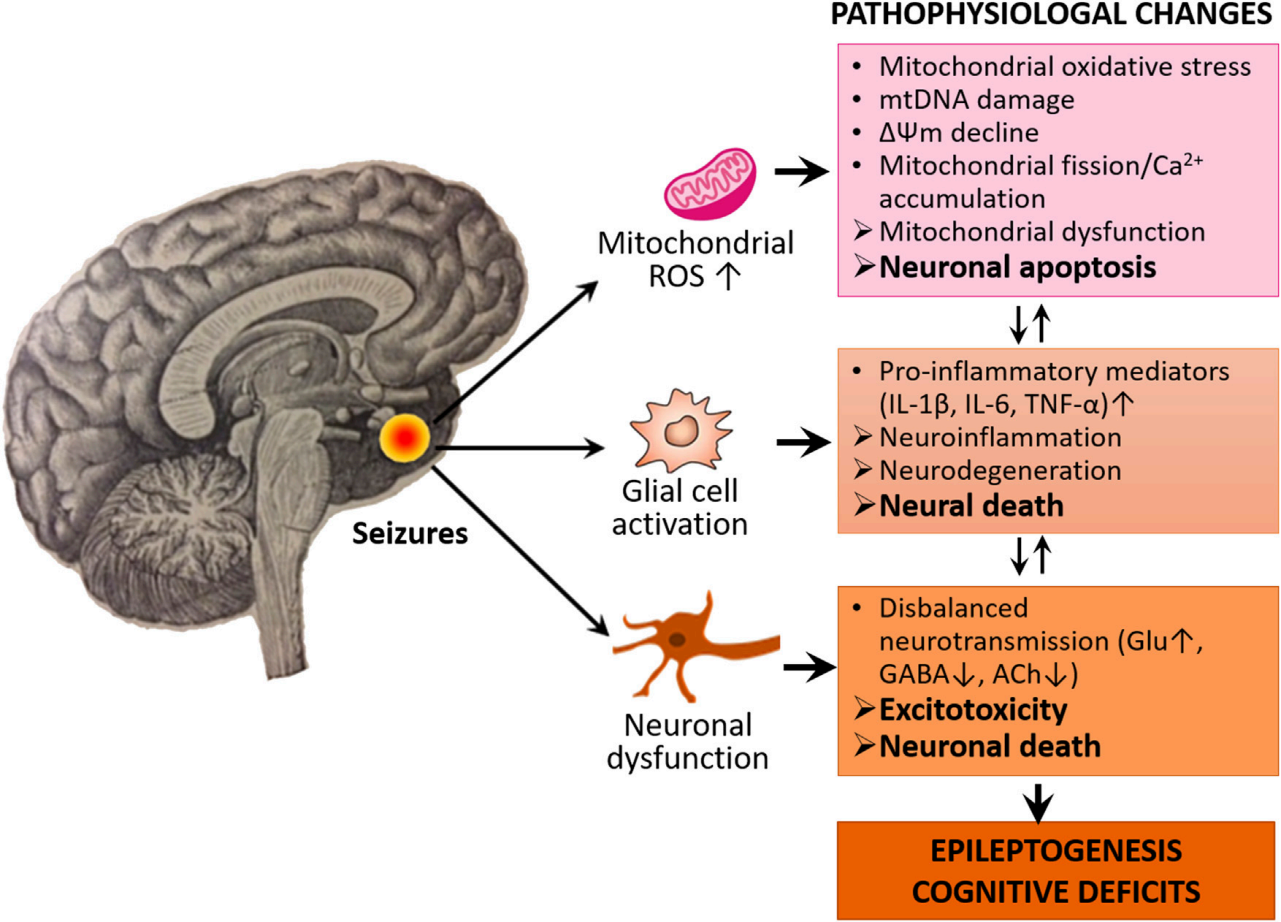
Oxidative stress, neuroinflammation, and excitotoxicity characterize the epileptogenic process and cognitive deficits. The activated neuroglia (microglia and astrocytes) releases pro-inflammatory mediators such as cytokines including IL-1β, IL-6, and TNF-α, thereby leading to neuroinflammation and neurodegeneration. In oxidative stress conditions, the mitochondria increase ROS production which causes mitochondrial oxidative stress, damage and dysfunction, thereby leading to neuronal apoptosis. Seizures also cause disturbance of neurotransmission which leads to disbalanced excitatory and inhibitory neurotransmission. There is also a causal relationship between neurodegeneration, apoptosis, and disbalance of neurotransmission, which eventually leads to epileptogenesis and associated cognitive deficits.

Neuronal mitochondria—the primary sites of reactive oxygen species (ROS) generation in the brain—are particularly sensitive and vulnerable to oxidant damage by ROS during epileptic seizures. Mitochondrial oxidative stress (MOS) caused by ROS overproduction is the main culprit that triggers diverse cell death pathways (Zhao et al., 2020). Several factors are likely to be implicated in the modulation of MOS, neuronal death, and epilepsy (Figure 1).

### 2.1 Apoptosis

The activation of both extrinsic and intrinsic apoptotic signaling pathways has been reported to exacerbate seizure-induced brain damage and leads to SE. An excessive and/or consistent neuronal excitation associated with seizures causes increased production of ROS, the liberation of cytochrome c from mitochondria, leading to activation of downstream caspases (e.g., caspase-3) and eventually neuronal apoptosis (Engel and Henshall, 2009).

### 2.2 mtDNA damage

Increased production of ROS by epileptic seizures also causes mtDNA damage that can disrupt normal functions of mitochondrial ETC thus reduce ATP supply to the neural tissue (Jarrett et al., 2008).

### 2.3 Mitochondrial fission

Seizures-induced oxidative stress accelerates mitochondrial fission, increases expressions of cytochrome c and caspase-3 as well as reduces neuronal viability (Qiu et al., 2013).

### 2.4 Mitochondrial Ca^2+^ accumulation

Seizure-induced stress on neurons enhances accumulation of mitochondrial Ca^2+^, which can not only further stimulate overproduction of ROS, but also facilitate the release of cytochrome c from the mitochondria to cytosol, triggering caspase-3-dependent neural apoptosis (Fricker et al., 2018; Ge and Wang, 2020).

### 2.5 Neuroinflammation

There is also evidence supporting the contribution of MOS to neuroinflammation in epilepsy. In various animal models of epileptogenesis, a typical neuroinflammatory response characterized by robust astrogliosis, microglial activation, and the production of cytokines and chemokines has been observed and associated with neuroinflammation (Pauletti et al., 2019; Vezzani, Balosso and Ravizza, 2019; Terrone et al., 2020).

These data suggest that MOS is likely an important pathogenic factor of epilepsy and targeting MOS and the intrinsic mitochondrial pathway might be a novel strategy to suppress neuronal apoptosis and alleviate epileptogenesis. In line with this assumption, Coenzyme Q10 (CoQ10)—an antioxidant produced naturally by our body—has evident neuroprotection against pentylenetetrazol (PTZ)-induced kindling *via* suppressions of oxidative damage and neuroinflammation (Bhardwaj and Kumar, 2016). Moreover, Co Q10 levels in epilepsy patients were significantly lower in epilepsy patients compared to healthy controls (Simani et al., 2020). To some extent, these data indirectly reflect the relationship between mitochondrial oxidative damage and neuroinflammation.

### 2.6 Excitotoxicity

Furthermore, oxidative stress and mitochondrial dysfunction contribute to glutamate-induced excitotoxicity and later neuronal apoptosis. The excitotoxicity–induced damage and death of hippocampal neurons are quite recurrent in cases of epilepsy, contributing to cognitive dysfunction (Sabogal-Guáqueta et al., 2019).

Taken together, these studies suggest that substances with antioxidant, anti-inflammatory, and anti-excitotoxic activities may promote neuroprotection and prevent epilepsy by reducing brain oxidative stress, neuroinflammation, neuronal apoptosis, and improving cognitive dysfunction.

## 3 The genus artemisia and its antiepileptic activity

The genus Artemisia, one of the most distributed genera in the Asteraceae family, is mostly distributed in Europe, Asia, and North America (Abad et al., 2012). Many of them have been used since ancient times as folklore remedies for various diseases, such as malaria, fever, cough, and gut parasitic diseases (Rasooli et al., 2003; Bora and Sharma, 2011b). Pharmacological studies demonstrated that Artemisia species possess a wide range of antioxidant, anti-inflammatory, antimalarial, antimicrobial, antiviral, antitumor, antipyretic, antihemorrhagic, anticoagulant, antianginal, antihepatitic, antiulcerogenic, and antispasmodic activities (Abad et al., 2012).

Despite their wide diversity, distribution, and application, most Artemisia species investigated to date have similar chemical compositions. The principal bioactive constituents found in this genus include isoprenoids, flavonoids, phenolic acids, coumarins, glycosides, sterols, polyacetylenes, and caffeoylquinic acids (Tan, Zheng and Tang, 1998; Bora and Sharma, 2011b), and the presence of these bioactive compounds in varying proportions in different plants might be attributed to Artemisia’s diverse pharmacological activities. For example, flavonoids, which are rich in *Artemisia annua* (Baraldi et al., 2008), possess numerous antioxidant, anti-inflammatory, and anti-apoptotic activities.

In recent years, more and more evidence has shown that oxidative stress and neuroinflammation play important roles in the pathophysiology of acquired epilepsy. A line of experimental studies has demonstrated that oxidative stress and neuroinflammation are implicated in the development of recurrent seizures and SE (Rowley et al., 2015; McElroy et al., 2017; Wang et al., 2017; Vezzani, Balosso and Ravizza, 2019) as well as cognitive abnormalities caused by recurrent seizures by inducing oxidative damage to neural tissue (Méndez-Armenta et al., 2014; Lee et al., 2017; Deng et al., 2019). Therefore, the focus of epilepsy research and treatment strategy has now shifted to seeking alternative approaches, such as herbal plants, with better efficacy and minimal side effects (Gao et al., 2014; Kwon et al., 2019; Rehman et al., 2019). It is postulated that the use of plant-based antioxidants (e.g., flavonoids) to reduce or eliminate neuroinflammation and oxidative damage might be a desirable strategy in the treatment of epilepsy and epilepsy-/AEDs-induced cognitive impairment.

Several Artemisia species have been used in folklore medicine to treat epileptic seizures, and the anticonvulsant efficacy of these plants was confirmed by *in vivo* animal experiments using acute and chronic epilepsy (De Lima, Morato and Takahashi, 1993; Sayyah, Nadjafnia and Kamalinejad, 2004; Mio et al., 2010; Khan et al., 2016; Kediso et al., 2021). Studies indicated that essential oil, crude extracts, and different fractionations of Artemisia species can prevent seizures. De Lima et al. (1993) reported earlier that the hydroalcoholic extract (HE) of *Artemisia verlotorum* prevented non-invasive electroshock (75 mA, 60 Hz) and the seizure-induced by pentylenetetrazole. In addition, HE also increased the latencies of seizures in both pilocarpine and 3-mercaptopropionic acid pilocarpine-induced mice (De Lima et al., 1993). These results suggest that extracts of A. verlotorum are able to protect against experimental convulsions elicited by various agents. *Artemisia dracunculus* L. is used as an antiepileptic remedy in Iranian folklore medicine. Essential oil of *A. dracunculus* L. demonstrated the anti-seizure activity in mouse models of acute epilepsy (Sayyah, Nadjafnia and Kamalinejad, 2004). The extracted *A. dracunculus* L. essential oil showed a dose and time-dependent anticonvulsant activity in both maximal electroshock (MES; ED50 0.84 ml/kg) and pentylenetetrazole (PTZ; ED50 0.26 ml/kg) mice models of seizure, respectively. After gas chromatography (GC)/mass spectrometry (MS) analysis of the essential oil, authors postulate that monoterpenoids in the essential oil may be responsible for the anticonvulsant effects (Sayyah, Nadjafnia and Kamalinejad, 2004). The potential anti-epileptic effect of *Artemisia copa Phil*. was explored by Mio et al. (2010) as a part of their psychopharmacological studies. They evaluated the anticonvulsant activity of plant water extracts on a PTZ-induced mouse model and found that *Artemisia copa Phil*. extracts (150 mg/kg) significantly increased the latency time and decreased the duration of seizures and mortality in mice. Khan et al. (2016) studied the anticonvulsive effect of *Artemisia Indica* fractions and the involvement of GABA-A receptors using electrophysiological methods. The results showed that the isolated carnosol, ursolic acid, and oleanolic acid (10–100 mg/kg) had significant anticonvulsant activity in a PTZ-induced epilepsy mouse model by positively regulating the α1β2γ2L GABA-A receptor activity. Kediso et al. (2021) evaluated the effect of an aqueous ethanolic extract of *Artemisia afra* on pentylenetetrazole-induced seizures in mice. They noted that extracts of *Artemisia afra* showed a delay in seizure onset and a reduction in the duration of convulsions in a dose-dependent manner.

To sum up, certain Artemisia species have been found to be effective anticonvulsants. However, the exact pharmacologically active compounds of the genus Artemisia and the underlying cellular and molecular mechanisms through which Artemisia exerts antiepileptic effects are not fully understood. There is increasing evidence suggesting that some Artemisia species might have therapeutic potential for epilepsy due to their antioxidant, anti-inflammatory, and anti-apoptotic properties as well as their regulation of neurotransmission (Hamsalakshmi et al., 2022).

## 4 Antiepileptic mechanisms of the genus artemisia

### 4.1 Antioxidant activity of the genus artemisia

Oxidative stress generated by ROS may play a role in the occurrence and development of epilepsy, and changes in mitochondria-related oxidative stress status can lead to neuronal apoptosis. Various plants from the Artemisia species demonstrate protective activities against oxidative damage to neural tissue *in vitro* and *in vivo*. Treatment with Artemisia extracts is able to protect neurons or recover the oxidative stress-induced damage by preventing the formation of free radicals, enhancing the activity of the endogenous antioxidant system, and regulating apoptosis signaling pathways.

One initial study with Artemisia extracts indicated that the methanol extract of *Artemisia absinthium* has free radical scavenging activity *in vitro* and antioxidant capacity in the animal brain (*in vivo*) by decreasing thiobarbituric acid reactive substances (TBARS) and restoring levels of superoxide dismutase (SOD) and glutathione (GSH) (Bora and Sharma, 2011a). Another recent study using rats showed that the levels of the pro-oxidants MDA (an indicator of lipid peroxidation) and nitric oxide (NO) were significantly reduced, while the levels of antioxidant enzymes GSH and its gene expression in cortical tissue were significantly increased in streptozotocin (STZ)-induced diabetic rats exposed to ethanol extract of *Aretemisia judaica* (Albasher et al., 2020). More recently, Rashidi et al. (2021) demonstrated that ethanolic extract of *Aretemisia absinthium* has antioxidant effect on 6-hydroxydopamine (6-OHDA)-induced oxidative stress in SH-SY5Y cells. The extract of the plant at concentrations ranging from 6.25 to 25 μg/ml has been found to significantly decrease intracellular ROS formation induced by 6-OHDA. The plant extract also increases the GSH level and SOD activity. Similarly, various fractions of two Artemisia species—*Aretemisia turanica* and *Aretemisia turcomanica*—were found to effectively suppress H_2_O_2_-induced oxidative stress and apoptosis of PC12 cells as well as restored the H_2_O_2_-induced GSH depletion (Hosseinzadeh et al., 2018). Besides the crude Artemisia extracts, the essential oil from *Aretemisia campestris* has been shown to be a potent antioxidant (Saoudi et al., 2017). Pretreatment of animals with the essential oil significantly reduces deltamethrin-induced oxidative damage in rat brain tissue by alleviating lipid peroxidation, oxidative stress, and degeneration of brain tissue (Saoudi et al., 2017).

The antioxidant activity of Artemisia was also investigated using secondary metabolites of the genus. Caffeoylquinic acids and their derivatives isolated from *Aretemisia princeps Pam*. showed a potent antioxidant effect on β -amyloid-induced oxidative stress in PC12 cells in a dose-dependent manner. Under oxidative stress conditions, PC12 cells were treated with whole extract and 3,5-dicaffeoylquinic acid (3,5-diCQA) increased the cell viability by approximately 1.6 and 2.4 times, respectively, compared to the control without treatments (Lee et al., 2011). In addition, 3,5-diCQA isolated from *Aretemisia argyi* H. also demonstrated potent antioxidant property (Kang et al., 2016). In this study, 3,5-diCQA could restore cognitive dysfunction and the antioxidant capacity in trimethyltin (TMT)-treated mice by reducing the amount of pro-oxidant malondialdehyde (MDA) and augmenting the levels of oxidized GSH in the brain tissue of ICR mice (Kang et al., 2016). Moreover, DSF-52, a sesquiterpene dimer isolated from *Aretemisia argyi*, could suppress NADPH oxidase blocking ROS production in lipopolysaccharide (LPS)-induced BV-2 cells (Zeng et al., 2014). Finally, *Aretemisia amygdalina* showed antioxidant action on differentiated N2a and SH-SY5Ycells (Sajjad et al., 2019). The oxidative stress and cell death induced by H_2_O_2_ in these cell lines were attenuated by different extracts of *Aretemisia amygdalina* by upregulation of the Nrf2 signaling pathway, which is known to be an emerging modulatory pathway of cells resistant to oxidants (Saha et al., 2022).

From the aforementioned data, it was suggested that the antioxidant properties and underlying mechanisms of the genus Artemisia might be attributed to the bioactive secondary metabolites of the plants. To date, most studies associated with the antioxidant activity of Artemisia extracts have focused on Artemisinin—the principal bioactive isoprenoids isolated from *Aretemisia annua*. A pioneering study conducted by Zheng et al. (2016) demonstrated that Artemisinin has a neuroprotective effect on sodium nitroprusside-induced oxidative damage to primary cortical neurons and PC12 cells *in vitro*. They found that pretreatment of PC12 cells with Artemisinin could protect cell viability by reducing oxidation and preventing the decline of mitochondrial membrane potential. In addition, they also demonstrated by Western blotting analysis that the neuroprotective effect of Artemisinin is associated with the activation of the extracellular regulated protein kinases (ERK) pathway, which is responsible for intracellular signaling. The involvement of the ERK/CREB signaling pathway was also supported by other studies (Chong and Zheng, 2016), which demonstrated that H_2_O_2_-induced oxidative damage can be suppressed by Artemisinin in retinal pigment epithelial cells D407 through restoring cell morphology, mitochondrial membrane potential, and slowing intracellular ROS generation. In line with those findings, Yan et al. (2017) demonstrated that Artemisinin has a protective function against H_2_O_2_-induced oxidative stress in retinal neuronal cells RGC-5. In this study, a decreased ROS accumulation and cell apoptosis, as well as an increased mitochondrial membrane potential has been observed following the treatment of cells with Artemisinin. The Western blotting analysis indicated that H_2_O_2_-induced oxidative stress in RGC-5 cells upregulated the phosphorylation of P38 and ERK1/2 kinase pathways, which could be reversed by Artemisinin. Interestingly, they also observed that intravenous administration of artemisinin in a concentration-dependent manner protected the retinal function from light-exposed damage by measuring electrical responses in retinal photoreceptors using flash electroretinography (Yan et al., 2017). The neuroprotective function of Artemisinin against glutamate-induced oxidative stress was also reported by Lin et al. (2018). They investigated the effects of artemisinin on a mouse hippocampal cell line (HT-22) damaged by glutamate-induced oxidative stress. The results demonstrated that the overproduction of ROS and the collapse of mitochondrial membrane potential caused by glutamate-induced oxidative stress could be rescued by the activation of the protein kinase B (Akt)/Bcl-2 signaling pathway in the cell line treated with artemisinin. In addition to artemisinin, administration of 3,5-diCQA, a phenolic compound extracted from *Artemisia argyi* H., prevented neuronal apoptosis from the mitochondrial pathway in the brain tissue of ICR mice exposed to TMT (Kang et al., 2016). In this study, 3,5-diCQA reduced TMT-induced mitochondrial ROS production and increased TMT-lowered mitochondrial membrane potential and ATP levels thus providing significant protection against TMT-induced mitochondrial dysfunction and cellular apoptosis (Kang et al., 2016). Phosphorylation of microtubule-associated protein tau (p-tau) by protein kinase B/Akt (Akt), plays an essential role in Akt-mediated anti-apoptotic signaling (Ksiezak-Reding et al., 2003). Kang et al. (2016) also found that treatment with 3,5-diCQA could increase the ratio of phosphorylated-Akt (p-Akt)/Akt, which was reduced in the TMT-treated mice, and decrease levels of phosphorylated tau (p-tau) and Bax, which were increased in the TMT-treated mice. Taken together, Artemisia extracts exert their antioxidant and anti-apoptotic effects by decreasing mitochondrial ROS generation, increasing antioxidant enzyme levels, recovering mitochondrial membrane potential, and regulating signaling pathways such as ERK/CREB and Akt/Bcl-2. These signaling pathways therefore could be potential targets for the treatment of epilepsy.

### 4.2 Anti-neuroinflammatory effects of the genus artemisia

Microglia-associated neuroinflammation is presumed to contribute to neuronal injury in various neurodegenerative disorders including epilepsy. Neuroinflammation in epilepsy is primarily characterized by robust astrogliosis, microglial activation, and the production of cytokines and chemokines (McElroy et al., 2017; Vezzani, Balosso and Ravizza, 2019). Several bioactive molecules isolated from Artemisia extract were able to significantly eliminate neuroinflammation in brain tissue and neural cell lines. Artemisinin B isolated from *Aretmisia annua* and DSF-52 isolated from *Artemisia argyi* were found to exhibit significant anti-neuroinflammatory effects on LPS-activated BV2 cells (microglial cell model) (Zeng et al., 2014; Qiang et al., 2018). Both artemisinin B and DSF-52 significantly downregulated LPS-induced increase in NO production, and gene expression levels of inflammatory cytokines IL-1β, IL-6, TNF-α, and upregulated gene expression levels of the anti-inflammatory cytokine IL-10 (Zeng et al., 2014; Qiang et al., 2018). DSF-52 also downregulated pro-inflammatory Prostaglandin E2 (PGE2), iNOS, COX-2 and granulocyte-macrophage colony-stimulating factor (GM-CSF) cytokines (Zeng et al., 2014). Another important finding of their studies is that both artemisinin B and DSF-52 inhibited major inflammatory transcription factor NF-κB in a dose-dependent manner (Zeng et al., 2014; Qiang et al., 2018). DSF-52 also inhibited the phosphorylation of NF-κB, IkB, and Akt, the activation and translocation of NF-kB from the cytoplasm into the nucleus, and NF-kB-DNA binding activity (Zeng et al., 2014). In addition to the Akt/IkB/NF-kB signaling pathway, DSF-52 also blocked JNK/p38 MAPKs and Jak2/Stat3 inflammatory signaling pathways by inhibiting the phosphorylation of respective molecules. Artemisinin B also reduced glial cell surface receptor TLR4 and its downstream adaptor protein MyD88 levels, which were abnormally high in the model group (Qiang et al., 2018). Both TLR4 and MyD88 are important because their association eventually leads to the activation of the NF-κB and MAPK signaling pathways and thus to the expression of inflammatory cytokines and chemokines (Esen and Kielian, 2006). Based on these findings, the study suggests that artemisinin B inhibits neuroinflammation *via* the TLR4-MyD88-NF-kB signaling pathway (Qiang et al., 2018). The ethanol extract of *Artemisia judaica* significantly reduced STZ-induced increase in the pro-inflammatory TNF-α and iNOS levels and expression in the cortical tissue of diabetic rats, comparable to the positive control metformin (Albasher et al., 2020). The study suggests that *Artemisia judaica* inhibits the development of cortical inflammation in diabetic rats (Albasher et al., 2020). In line with those findings, Artemisinin isolated from *Artemisia annua* significantly reduced the immunoreactivity of glial cells, as detected by a decrease in GFAP and Iba1 markers, cleaved caspase 1, and IL-1β levels, as well as restored microglia morphology in the cerebral cortex region of 3xTg AD mouse model (Zhao et al., 2020).

In addition to Artemesinin, the anticonvulsant effects of some other anti-inflammatory flavonoids isolated from the genus Artemisia have been confirmed in various experimental models of epilepsy, and their mechanisms of action have been explored. Daneshkhah and Setorki. (2019) showed that the anticonvulsant activity of essential oil (EO) of *Artemisia persica* significantly decreased seizure latency and tonic seizure in PTZ-kindled mice by reducing brain inflammation *via* downregulation of the expression of pro-inflammatory cytokines (IL-1β and TNF-α). Golechha et al. (2011) observed that pretreatment of kainic acid (KA)-induced epileptic rats with naringin significantly attenuated seizures and cognitive deficits by inhibiting the production of the pro-inflammatory cytokine TNF-α in the brain. Hispidulin—a flavonoid compound found in *Artemisia abrotanum* L. and *Artemisia herba-alba*—substantially attenuated kainic acid-induced seizures and hippocampal neuronal cell death by the suppression of microglial activation and the production of pro-inflammatory cytokines IL-1β, IL-6, and TNF-α (Lin et al., 2015). Zhu et al. (2019) indicated that isoliquiritigenin pretreatment of kainic acid-induced epileptic rats has shown neuroprotective and anti-inflammatory effects *via* the TLR4/MYD88 signaling pathway. These studies suggest that the genus Artemisia has antiepileptic therapeutic potential, at least in part because it contains flavonoid compounds with promising antioxidant and anti-inflammatory properties.

### 4.3 Anti-apoptotic and neuroprotective effects of the genus artemisia

Recently, several studies have documented the neuroprotective and antiapoptotic effects of Artemisia. As we mentioned in the previous section (Section 4.1), 3,5-diCQA isolated from *Artemisia argyi* demonstrated significant protection against neuronal apoptosis and cognitive function *via* the mitochondrial apoptotic pathway (Kang et al., 2016). Kwon et al. (2018) reported the neuroprotective effect of *Artemisia capillaris* and the possible involvement of apoptotic mechanisms. They showed that the ethanol extract of *A. capillaris* rescued BCCAO-induced neuronal degeneration and apoptosis, accompanied by an inhibition of BCCAO-induced increase of caspase-3-positive cells in the mouse hippocampus (Kwon et al., 2018). The ethanol extract of *A. judaica* increased the level of anti-apoptotic marker Bcl2 and decreased the level of pro-apoptotic marker Bax by blocking STZ injection-induced neuronal apoptosis in rat brains (Albasher et al., 2020). Artemisinin isolated from *A. annua* was found to reduce p-tau levels and protect brain tissue in 3xTg mice and SH-SY5Y cells *in vitro* (Zhao et al., 2020). In the brain tissue of 3xTg mice, Artemisinin treatment significantly reduced Aβ plaques in the cerebral cortex and hippocampus, damage to neurons and Nissl bodies, and the number of apoptotic cortical neurons. Further investigations indicated that p-ERK1/2, P-CREB levels, and Bcl-2/Bax ratio were significantly increased in the artemisinin-treated 3xTg animal group compared to the non-treated 3xTg group. In the SH-SY5Y cell line, pre-treatment with artemisinin prevented the Aβ (1–42)-induced SH-SY5Y cell death in a dose-dependent manner by protecting the mitochondrial membrane potential and decreasing cellular ROS production (Zhao et al., 2020). Moreover, artemisinin treatment resulted in a time- and dose-dependent increase in phosphorylated ERK1/2 and CREB levels in SH-SY5Y cells. Taken together, this study suggests that artemisinin exerts a potential anti-apoptotic effect on neural cells by regulating the ERK/p-CREB/Bcl2 pathway.

### 4.4 Beneficial effect of artemisia on cognitive function

Cognitive abnormality, especially impairment in learning and memory, is one of the serious comorbidities of epilepsy that has a significant negative impact on patients’ quality of life (Holmes, 2015). Despite some reports, the mechanisms underlying epilepsy-associated cognitive impairment have not been completely elucidated. According to the literature, it is likely that many factors are involved, including the dysregulation of neurotransmitter systems (cholinergic, glutamatergic, GABAergic), microglial activation and neuroinflammation, and oxidative stress, which ultimately result in neural tissue damage and cognitive impairment. Several Artemisia species (some of them mentioned in previous sections) have been identified as having neuroprotective potential and the capacity of restoring cognitive impairment by suppressing oxidative stress, neuroinflammation, and regulating the cholinergic neurotransmitter system. For instance, methanol extract of *Artemisia absinthium* significantly reduced cerebral infarct volume and short-term memory impairment in a mouse model of cerebral ischemia induced by middle cerebral artery occlusion (MCAO) *via* regulation of oxidative stress/antioxidant parameters (Bora and Sharma, 2010). In this study, Artemisia treatment resumed levels of antioxidant enzymes (GSH, SOD, and CAT) that were decreased in the brain of the MCAO model, and the concentration of the thiobarbituric acid reactive substance (TBARS)—a lipid peroxidation marker—that was increased in the brain of MCAO model (Bora and Sharma, 2010). Interestingly, administration of the plant extract before focal cerebral ischemia also prevented short-term memory impairment suggesting that *A. absinthium* can not only restore already damaged neural tissue but also protect them from damage (Bora and Sharma, 2010). In another study using a mouse model of Alzheimer’s disease established by intra-cerebroventricular injection of the toxic fragment of Aβ (Aβ25-35), artemisinin B—a type of artemisinins derived from *A. annua*—significantly ameliorated learning and memory of this mouse model in water-maze and step-through tasks. In the same model group, artemisinin B also inhibited the activation of microglia as well as the loss of the Nissl bodies and synaptophysin in the CA1 neurons of the hippocampus (Qiang et al., 2018). These studies indicated that oxidative stress, microglial activation, and neuroinflammation are involved in neuronal damage in the hippocampus, and Artemisia extracts could protect and restore memory deficiency by suppressing those processes. The genus Artemisia species can also restore cognitive impairment by modulating neurotransmitter and neuromodulator activity. 3,5-diCQA pretreatment significantly inhibited TMT-induced disruption of the cholinergic neurotransmission and cognitive impairment by increasing acetylcholine (ACh) levels and decreasing acetylcholinesterase (AChE) activity in the brain tissue of ICR mice compared to negative controls (Kang et al., 2016). In line with these findings, Kwon et al. (2018) reported that treatment with ethanol extracts of *A. capillaries* could recover BCCAO-mediated neurodegeneration and cognitive impairment in mice by stimulating nicotinic acetylcholine receptors (nAChRs) and inhibiting AChE activity. They showed that the nonselective neuronal nicotinic receptor antagonist mecamylamine markedly blocked the neuroprotective effect of *A. capillaris* (Kwon et al., 2018). The authors hypothesize that the plant metabolites might activate the PI3K-Akt signaling cascade, which leads to the activation of neuronal nAChRs (Kwon et al., 2018). Hence, the study suggests that the neuroprotective and pro-cognitive action of *A. capillaris* is due to the activation of the cholinergic neurotransmitter system in the brain.

**FIGURE 2:**
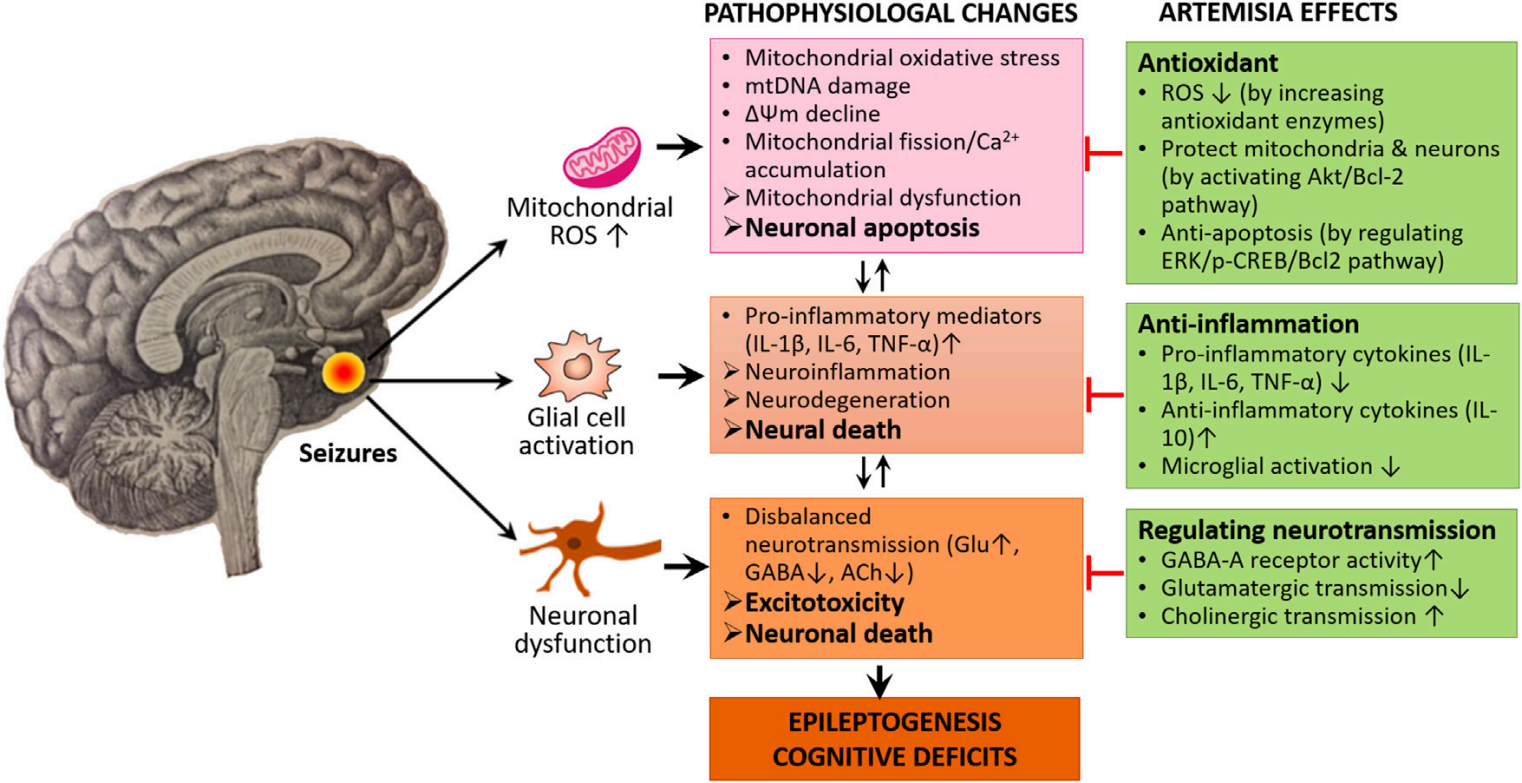
Neuroprotective mechanisms of Artemisia. Artemisia extracts exert their neuroprotective and anti-epileptogenic effects through the modulation of molecular signaling pathways involving ROS production and neuroinflammation as well as regulating disbalanced neurotransmission, thereby preventing excess neural death and apoptosis.

These data altogether conclude that MOS, mitochondrial dysfunction, neuroinflammation, disbalanced neurotransmission, and ultimately neuronal death/apoptosis could be also implicated in epileptogenesis and cognitive impairment. We assume that targeting these processes using Artemisia extracts have promising protective roles against the seizure generation, epileptogenesis, and cognitive comorbidities of epilepsy (Figure 2).

## 5 Discussion

Epilepsy is defined as one of the World Health Organization (WHO) public health priorities for its effective care, prevention, and treatment. It is a potentially life-threatening disease and associated with increased disability and mortality (Thurman et al., 2018). Although AEDs are the primary treatment method for controlling epilepsy, most of these drugs are considered to be associated with temporary seizure control, drug resistance, and cognitive abnormalities (Sørensen and Kokaia, 2013). Growing evidence reveals that oxidative stress, neuroinflammation, and dysregulated neurotransmission contribute to the pathophysiology of epilepsy and cognitive impairment. Neuronal damage and death/apoptosis are most likely to be directly or indirectly caused by neuronal hyperexcitability, oxidative stress, and neuroinflammation. Therefore, any antioxidant, anti-inflammatory, and neuroprotective interventions might prevent epileptogenesis and its cognitive comorbidities.

Nowadays, the growing need for more natural sources of medicine and certain prominent pharmacological activities of Artemisinin has driven scientific interest in Artemisia species. In recent years, a series of experimental studies have demonstrated that certain genus Artemisia has anticonvulsant effects in animal models of epilepsy *in vivo* (Table 1) and antioxidant, anti-neuroinflammatory, and neuroprotective effects in neural cell lines *in vitro* (Tables 2, 3), in which oxidative stress were induced and epileptic seizures were developed.

**TABLE 1:** Anticonvulsant activities of Artemisia species *in vivo* in animal models.

Species	Material	Model	Dose	Authors
*Artemisia verlotorum*	hydroalcoholic extract	PTZ, 3-MP- induced mice	2 g/kg	De Lima, Morato and Takahashi, (1993)
*Artemisia dracunculus* L	Essential oil	MES-induced mice	0.84 ml/kg	Sayyah, Nadjafnia and Kamalinejad, (2004)
		PTZ-induced mice	0.26 ml/kg	
*Artemisia copa Phil*	Water extracts	PTZ-induced Mice	150 mg/kg	Mio et al. (2010)
*Artemisia Indica* Lin	Carnosol, ursolic acid, and oleanolic acid compounds	PTZ-induced mice	10–100 mg/kg	Khan et al. (2016)
*Artemisia. afra*	Hydroethanolic extract	PTZ-induced mice	250–1,000 mg/kg	Kediso et al. (2021)

**TABLE 2:** Antioxidant, anti-inflammatory, and neuroprotective effects of Artemisia species *in vivo* and its mechanisms of action.

Species	Compounds	Models	Mechanisms	References
*Artemisia absinthium*	Methanolic extract	Cerebral I/R-induced mice	Restore SOD and GSH levels and decrease TBARS	Bora and Sharma, (2011a)
	3,5-diCQA	TMT-induced cognitive dysfunction mice	Increasing the GSH ratio. Protection of mitochondrial activities and the repression of apoptotic signaling molecules such as p-Akt, BAX, and p-tau (Ser 404)	Kang et al. (2016)
*Artemisia capillaries*	Ethanol extracts	Ischemic brain injury-induced mice	Activation of nicotinic acetylcholine receptors	Kwon et al. (2018)
*Artemisia campestris*	Essential oil	Brain tissue of deltamethrin treated rats	Change antioxidant enzymes level	Saoudi et al. (2017)
*Artemisia judaica*	Ethanol extracts	STZ-induced diabetic rats	Improvement of gene expression of the antioxidant enzymes. Decreasing the level and expression of TNF-α and iNOS in diabetic rats	Albasher et al. (2020)

**TABLE 3:** Antioxidant, anti-inflammatory, and neuroprotective effects of Artemisia species *in vitro* and its mechanisms of action.

Species	Compounds	Models	Mechanisms	References
*Artemisia absinthium*	Ethanolic extract	6-OHDA-induced SH-SY5Y cells	increases the GSH level and SOD activity. decrease intracellular ROS	Rashidi et al. (2021)
*Artemisia princeps Pampanini*	Phenolics	PC12 cells	Decreasing intracellular oxidative stress	Lee et al. (2011)
*Artemisia argyi*	DSF-52	LPS-mediated BV-2 microglial	Suppression of NF-κB, JNK/p38 MAPKs, and Jak2/Stat3 signaling pathways. Reduction of the expression levels of the inflammatory cytokines IL-1β, IL-6, and TNF-α	Zeng et al. (2014)
*Artemisia annua*	Artemisinin	PC12 cells	Activation of ERK pathway	Zheng et al. (2016)
		D407 retinal pigment epithelial cells	Activation of ERK/CREB signaling pathway	Chong and Zheng, (2016)
		Neuronal cells RGC-5	Activation of P38 and ERK1/2 kinase pathway	Yan et al. (2017)
		LPS-mediated BV-2 microglial	Reduction of the expression levels of the inflammatory cytokines IL-1β, IL-6, and TNF-α. Inhibition of TLR4-MyD88-NF-kB signaling pathway	Qiang et al. (2018)
		HT-22 cells	Activation of kinase B (Akt)/Bcl-2 pathway	Lin et al. (2018)
		SH-SY5Y	activation of the ERK/CREB pathway and inhibition of apoptosis pathway	Zhao et al. (2020)
*Artemisia turanica*	Crude extracts	PC12 cells	Reduction of caspase-3 activity	Hosseinzadeh et al. (2018)
*Artemisia amygdalin*	Methanol, aqueous, and ethyl acetate extracts	Differentiated N2a and SH-SY5Ycells	Upregulate the Nrf2 signaling pathway	Sajjad et al. (2019)

However, the exact pharmacologically active compounds of Artemisia (except artemisinin) and the underlying mechanisms through which those bioactive compounds exert antiepileptic effects have not been extensively investigated. Based on all the data included and analyzed in the review, we assume that genus Artemisia subspecies possess potential antiepileptic and neuroprotective properties thanks to their antioxidant, anti-inflammatory, neurotransmitter regulating, and anti-apoptotic activities. The bioactive secondary metabolites of the genus Artemisia plant are likely to act through modulating multiple signaling pathways, such as extracellular regulated protein kinases (ERK/CREB/Bcl-2) pathway and intracellular (mitochondrial) pathway, as well as an emerging nuclear factor erythroid 2-related factor 2 (Nrf2) signaling pathway.

Therefore, the use of natural bioactive compounds isolated from Artemisia as potential pharmacological modulators targeting the aforementioned signaling pathways might be an alternative option for the treatment of epilepsy and its cognitive comorbidities. However, although many experimental studies have demonstrated anti-epileptic and neuroprotective activities of Artemisia species, most of them have emphasized crude plant extracts or fractions, and only a few pure bioactive compounds have been used in the studies (see Tables 1–3). In addition, there are other pitfalls in using Artemisia: long-term and low-dose Artemisia exposure may induce free radical scavengers that disrupt the endoperoxide bridge structure of Artemisia. Moreover, unexpected metabolic dysfunction or other abnormalities may occur after excessive use of Artemisia, including neurotoxicity and/or sperm DNA damage-induced genotoxicity (Singh, Giri and Giri, 2015; Sun and Zhou, 2017) Therefore, to ensure safety at appropriate doses, long-term testing should be evaluated. Finally, there is currently no preclinical and clinical trial evidence showing that Artemisia has a therapeutic effect on epilepsy.

## 6 Conclusion

This review further highlights the roles of oxidative stress, neuroinflammation, and excitotoxicity in seizure-mediated neuronal damage and apoptosis, as well as their possible contributions to epileptogenesis and cognitive impairment. It also confirms that the genus Artemisia possesses neuroprotective and antiepileptic potentials, which stem from their antioxidant, anti-inflammatory, neurotransmitter-modulating, anti-apoptotic properties by modulating oxidative stress caused by mitochondrial ROS production and an imbalance of antioxidant enzymes, by protecting mitochondrial membrane potential required for ATP production, by upregulating GABA-A receptor and nACh receptor activities, and by interfering with various anti-inflammatory and anti-apoptotic signaling pathways.

Although some fundamental research results have been achieved, there is still a lot of work to be done for further research on Artemisia and its neuroprotective effects. Firstly, many Artemisia species have not been assessed biologically and pharmacologically for their neuroprotective and antiepileptic activities, which requires further explorations in ongoing research. Secondly, although many experimental studies have demonstrated anti-epileptic and neuroprotective activities of the genus Artemisia species, most of the studies refer to crude extracts containing a cocktail of compounds. Only countable pure isolated compounds have been evaluated in the previous studies. Therefore, it needs more pharmacological studies using pure bioactive compounds to confirm the molecular mechanisms of Artemisia action. Thirdly, despite several secondary metabolites of the genus Artemisia having been shown to possess antiepileptic and neuroprotective properties in experimental conditions, bioavailability and BBB penetration require more *in vivo* animal experiments and clinical evaluation. Despite some unravelled mysteries that remain to be clarified in the future, the antioxidant and anti-inflammatory compounds in the genus Artemisia plants are undoubtedly favourable neuroprotective agents for the improvement of brain damage in patients with epilepsy. As the investigation continues, the genus Artemisia might prove to be a rich source of bioactive compounds needed for the development of new neuroprotective chemotherapeutical agents that alleviate neural tissue damage in patients with epilepsy and other neurodegenerative disorders. Their extracted and isolated bioactive compounds, if not toxic, can also serve as food additives for the prevention of oxidative stress-related disorders.

## Abbreviations

6-OHDA, 6-hydroxydopamine; ACh, Acetylcholine; AChE, Acetylcholinesterase; AED, Anti-epileptics drugs; Apaf-1, Apoptotic protease activating factor 1; DiCQA, Dicaffeoylquinic acid; GC/MS, Gas chromatography/mass spectrometry; GMSCF, Granulocyte-macrophage colony-stimulating factor; GSH, Glutathione; HE, Hydroalcoholic extract; ILAE, International League Against Epilepsy; MCAO, Middle cerebral artery occlusion; MES, Maximal electroshock; MOS, Mitochondrial oxidative stress; NO, Nitric oxide; Nrf2, Nuclear factor erythroid 2-related factor 2; OS, Oxidative stress; PGE2, Prostaglandin E2; p-tau, Phosphorylated tau; PTZ, Pentylenetetrazole; ROS, Reactive oxygen species; SE, Status epilepticus; SOD, Superoxide dismutase; TBARS, Thiobarbituric acid reactive substance; TMT, Trimethyltin.
